# Genetic Variations Associated with Long-Term Treatment Response in Bipolar Depression

**DOI:** 10.3390/genes12081259

**Published:** 2021-08-18

**Authors:** Gerard Anmella, Silvia Vilches, Jordi Espadaler-Mazo, Andrea Murru, Isabella Pacchiarotti, Miquel Tuson, Marina Garriga, Eva Solé, Mercè Brat, Giovanna Fico, Eduard Vieta

**Affiliations:** 1 Bipolar and Depressive Disorders Unit, Institute of Neuroscience, Hospital Clinic, University of Barcelona, IDIBAPS, CIBERSAM, 170 Villarroel st, 12-0, 08036 Barcelona, Catalonia, Spain; anmella@clinic.cat (G.A.); amurru@clinic.cat (A.M.); pacchiar@clinic.cat (I.P.); mgarriga@clinic.cat (M.G.); evsole@clinic.cat (E.S.); brat@clinic.cat (M.B.); gfico@clinic.cat (G.F.); 2 AB-Biotics, S.A., Av. de la Torre Blanca 57, 08172 Sant Cugat del Valles, Barcelona, Catalonia, Spain; vilches@ab-biotics.com (S.V.); espadaler@ab-biotics.com (J.E.-M.); tuson@ab-biotics.com (M.T.)

**Keywords:** treatment response, pharmacogenetics, precision medicine, genetic variants, bipolar disorder, psychiatric disorders

## Abstract

Several pharmacogenetic-based decision support tools for psychoactive medication selection are available. However, the scientific evidence of the gene-drug pairs analyzed is mainly based on pharmacogenetic studies in patients with major depression or schizophrenia, and their clinical utility is mostly assessed in major depression. This study aimed at evaluating the impact of individual genes, with pharmacogenetic relevance in other psychiatric conditions, in the response to treatment in bipolar depression. Seventy-six patients diagnosed with bipolar disorder and an index major depressive episode were included in an observational retrospective study. Sociodemographic and clinical data were collected, and all patients were genotyped using a commercial multigene pharmacogenomic-based tool (Neuropharmagen^®^, AB-Biotics S.A., Barcelona, Spain). Multiple linear regression was used to identify pharmacogenetic and clinical predictors of efficacy and tolerability of medications. The pharmacogenetic variables response to serotonin-norepinephrine reuptake inhibitors (SNRIs) (*ABCB1*) and reduced metabolism of quetiapine (*CYP3A4*) predicted patient response to these medications, respectively. *ABCB1* was also linked to the tolerability of SNRIs. An mTOR-related multigenic predictor was also associated with a lower number of adverse effects when including switch and autolytical ideation. Our results suggest that the predictors identified could be useful to guide the pharmacological treatment in bipolar disorder. Additional clinical studies are necessary to confirm these findings.

## 1. Introduction

Bipolar disorder (BD) is a complex and severe psychiatric condition characterized by biphasic mood episodes of mania or hypomania and depression, which are expressed as recurrent episodes of changes in energy levels and behavior that last from days to weeks, each with subsyndromal symptoms commonly present between major episodes [1]. The total lifetime prevalence of BD is approximately 2.4% [2]. Despite an apparent lower prevalence than unipolar depression [3], BD is associated with higher levels of functional impairment and compromised quality of life, posing a greater socioeconomic burden [4]. With a typical age at onset between late adolescence and early adulthood, BD represents one of the leading causes of disability among young people [5,6]. Regarding gender distribution, while BD type I is considered to affect men and women equally, BD type II has been more frequently reported among women [7]. Due to the progressive and debilitating course of this disorder, an early and effective therapeutic approach is key for patient prognosis [8,9].

Pharmacological intervention, combined with psychotherapy and psychoeducation, are the therapeutic tools for managing BD. The choice of treatment options is guided by the phase of illness (mania/hypomania/depression/mixed) and past treatment history. Therefore, treatment guidelines include the use of mood stabilizers (lithium and some anticonvulsants such as carbamazepine and valproate) but also a variety of other psychoactive drugs, including antipsychotics, antidepressants, anxiolytics, and combinations thereof [10,11,12,13,14,15]. However, treatment response is often inadequate, and the rate of remission, particularly in patients in a depressive episode, is low. This leads to multiple unsuccessful drug trials for many patients, with the probability of achieving remission decreasing with every additional treatment line. In addition, common side effects and poor tolerability to treatment are usually observed, particularly when multimodal drug therapy is used.

Patient’s characteristics impacting drug response include endogenous and environmental factors, such as age, sex, body mass index, organ function, disease, concomitant medications, or lifestyle. Variability in treatment efficacy and tolerability has also been shown to be influenced by inherited genetic variation. Common genetic variation has been estimated to explain up to 42% of the variance in antidepressant response [16], and several genes with polymorphisms of pharmacogenetic (PGx) relevance in the treatment of major depression have been identified [17], although genome-wide association studies (GWAS) suggest no single polymorphisms of large effect [18].

The effect of genetic variants in BD has been understudied in comparison with major depression, with lithium being the medication most extensively investigated [19]. The largest GWAS to date in BD suggests that 15 genes robustly linked to BD encode druggable targets such as HTR6—a serotonin receptor targeted by antidepressants and antipsychotics [20]. Another important GWAS on lithium response, conducted by the International Consortium on Lithium Genetics (ConLiGen) in 2563 patients with BD, found a significant association with a single locus of four linked single nucleotide polymorphisms (SNPs) on chromosome 21, which contained two genes coding for long non-coding RNAs of unknown function [21]. Remarkably, an independent prospective study with 73 participants treated with lithium monotherapy showed an association of this region with the rate of relapse in the two-year follow-up [21]. Important PGx progress has also been made in the context of severe life-threatening cutaneous drug reactions observed with antiepileptic mood-stabilizers. Carriers of the human leukocyte antigen HLA-B*15:02 allele who are of Asian descent are at an increased risk of developing Stevens-Johnson syndrome (SJS) and toxic epidermal necrolysis (TEN) [22,23]. Likewise, the presence of the HLA-A*31:01 allele has been associated with carbamazepine-induced hypersensitivity reactions in individuals of European, Korean, and Japanese ancestry [24]. Therefore, recommendations for HLA-B*15:02 and/or HLA-A*31:01 are included in the summary of products characteristics (SmPC) of carbamazepine, as well as in guidelines by the Clinical Pharmacogenetics Implementation Consortium (CPIC) or the Canadian Pharmacogenomics Network for Drug Safety (CPNDS) [25,26]. Another interesting example is the cytochrome (CYP) P450 superfamily, which includes the major drug-metabolizing enzymes involved in phase I reactions. Even though specific studies in BD are generally lacking, most drugs used to treat BD are metabolized by CYP450 enzymes. Therefore, genotyping the *CYP450* genes to characterize the metabolizer profile of a patient becomes a potentially useful tool for medication selection and dose adjustment [27].

Neuropharmagen^®^ (AB-Biotics S.A., Barcelona, Spain) is a commercial PGx-based tool that generates psychopharmacological recommendations based on pharmacogenetic guidelines issued by the CPIC and other reference organizations, PGx information included in the SmPC approved by regulatory agencies, and additional relevant information from selected clinical studies. The psychopharmacological recommendations are displayed using a safety-first approach that grades genetic polymorphisms associated with risk of adverse effects above the genetically determined metabolizer status and genetic variants of efficacy.

The clinical utility of Neuropharmagen^®^ in major depression was initially evaluated in a twelve-week randomized controlled trial (RCT) in Spain, including 316 participants with major depressive disorder (MDD). In this trial, the PGx-guided arm showed a higher responder rate when compared to treatment as usual (TAU), with the PGx-guided treatment effects being more consistent in patients with 1–3 failed drug trials, as well as higher odds of achieving better tolerability [28]. A reanalysis of this study showed that PGx-guided treatment was mostly beneficial in non-elderly subjects, and in those with moderate-to-severe depression, but not in patients with mild depression [28]. These results were further replicated in an eight-week, industry-independent RCT in South Korea, including 100 adult patients with MDD, in which the PGx-guided arm showed higher response rates, less depressive symptoms, and better tolerability of treatments compared to TAU [29].

The output of multigene PGx-based tools is based on the integrated analysis of several genes which could differ in their individual clinical utility in a disorder-related manner. Two small pilot trials have suggested a potential clinical utility of Neuropharmagen^®^ in patients with BD. A three-month prospective, observational trial including 30 patients with BD suggested that at baseline less than 5% of patients had an optimal treatment according to the PGx-guided information provided by Neuropharmagen^®^. The evolution of severity (as assessed by the Clinical Global Impression of Severity, CGI-S) displayed a statistically significant treatment x time interaction, favoring those patients whose therapy had been changed following the test recommendations. Additionally, after the three-month follow-up period, the percentage of adverse effects was reduced when prescribing treatments concordant with the test [30]. The second pilot study with Neuropharmagen^®^ in a BD cohort consisted of a 2-year mirror analysis of the expenditure of 30 patients with BD. Comparison of the expenditure before and after the change of therapy in agreement with the Neuropharmagen^®^ recommendations resulted in cost savings in terms of the number of consultations to the emergency room, and the number and mean duration of hospitalizations [31].

The aim of the present pilot study was to objectively evaluate the long-term impact (versus the typical 8–12-week follow-up periods) of the genetic variation in individual genes—already demonstrated to be relevant in the pharmacological treatment of other psychiatric conditions such as MDD or schizophrenia—on the therapeutic response and side effects profile in a cohort of well-characterized patients with bipolar depression, using Neuropharmagen^®^.

## 2. Materials and Methods

### 2.1. Study Design

**Figure 1:**
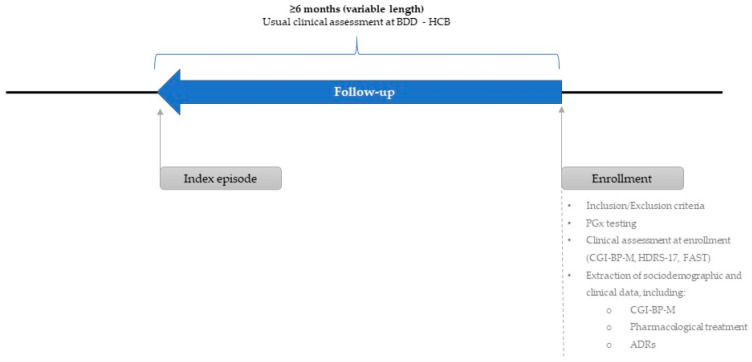
Schematic of the PGx-BP study procedures. BDD–HCB: Bipolar and Depressive Disorders Unit Program of the Psychiatry Service of the Hospital Clínic de Barcelona; CGI-BP-M: Clinical Global Impression for Bipolar Disorder, Modified version; FAST: Functioning Assessment Short Test; HDRS-17: 17-Item Hamilton Depression Rating Scale; PGx: Pharmacogenetic.

This observational, retrospective, epidemiological study enrolled a total of 76 patients from 1 March 2016 to 31 March 2016, among those attending the Bipolar and Depressive Disorders Unit Program of the Psychiatry Service of the Hospital Clinic de Barcelona (Barcelona, Spain). The follow-up was at least six months from the beginning of the index episode (IE), and the frequency of visits was determined by the usual practice at the Bipolar and Depressive Disorders Unit Program. Figure 1 depicts a schematic view of the study procedures.

The study was conducted in compliance with Good Clinical Practices (as described in the Guidelines for Good Pharmacoepidemiology Practices (GPP) of the International Society for Pharmacoepidemiology, 2008 review) and the guidelines of the Declaration of Helsinki. The study was approved by the Institutional Review Board (IRB) of Hospital Clínic de Barcelona, Spain (protocol code: PGx-BP; approval number and date: HCB/2015/0990, 4 January 2016), and the protocol was retrospectively registered at ClinicalTrials.gov as NCT04923204. All patients provided written informed consent to participate. In the case of disabled patients, informed consent was provided by the legal representative or responsible family.

### 2.2. Subjects

The study included patients 18 years and older, with a diagnosis of BD with an IE of major depression with or without associated psychotic symptoms—according to the Diagnostic Manual of Mental Disorder 4th Edition Text Revision (DSM-IV-TR). Patients undergoing electroconvulsive therapy (ECT) or with any serious or terminal medical organic disease or an intelligence quotient <85 were excluded.

### 2.3. Data Collection

The data were retrospectively collected from the patient clinical records of the hospital and added into an anonymized electronic database.

Sociodemographic and clinical data were extracted from all enrolled subjects, including the pharmacological treatment in the IE and mood switch during the IE—the latter for those patients applicable—and the presence and type of side effect associated with the pharmacological treatment in the IE. The adverse events present in the reviewed patients’ charts were as follows: weight gain, dyslipidemia (cholesterol, LDL, HDL, triglycerides), glucose disturbance, sedation, extrapyramidal symptoms, seizures, neuroleptic malignant syndrome, QTc prolongation, sexual dysfunction, and hyperprolactinemia. Other data recorded were gender, age, ethnicity, marital status, academic level, weight and height, psychiatric and/or non-psychiatric comorbidities, smoking, alcohol intake, substance use disorder, psychiatric family history, type of bipolar disorder (I, II, or not otherwise specified—NOS), time since diagnosis, date and duration of IE, treatments prior to IE, bipolar episodes (type and number), need of hospitalization in IE, psychosis, suicidal ideation and/or suicidal attempt, use of anxiolytics, presence of mood switch during the IE, and time to mood switch.

The patient’s clinical status was assessed by the treating psychiatrist according to the modified version of the Clinical Global Impression for Bipolar Disorder (CGI-BP-M) [32] at the beginning of the IE of major depression, at the end of the IE, and at enrolment. The 17-item Hamilton Depression Rating Scale (HDRS-17) [33] and Functioning Assessment Short Test (FAST) [34] were also administered at enrolment.

### 2.4. Genotyping and Reporting of Results

At enrolment, saliva samples from patients who gave their informed consent were collected with the commercial kit OG-510 (DNA Genotek Inc., Ottawa, ON, Canada) and sent to the laboratory of AB-Biotics S.A. (Barcelona, Spain) for DNA isolation and subsequent genotyping of selected variants. Details on the pharmacogenetic analysis have been previously described [28].

Briefly, Neuropharmagen^®^ provided an interpretive report assigning one or more colors to each drug depending on the subject’s genotype: green for the presence of pharmacogenetic variants associated with increased positive response or lower likelihood of side effects, amber for variants associated with altered metabolism or reduced efficacy, red for variants associated with side effects not related to reduced plasma clearance, and white (or “standard”) when none of the genetic variants analyzed for the said drug were detected (Figure S1 and Table S1). The Neuropharmagen^®^ results were used for the purpose of the current retrospective association study.

### 2.5. Statistics

Continuous variables were summarized as the means and standard deviations or median and range, while categorical variables were summarized as percentages. Because several genes, as well as clinical variables, may contribute to a given clinical outcome, the effect of the individual gene-drug pairs together with several clinical variables was analyzed by means of multivariate linear regression for continuous outcomes and multivariate binary logistic regression for dichotomous outcomes. A forward-selection approach with a *p*-value cutoff of 0.05 was used to identify the pharmacogenetic and clinical predictor variables with a significant contribution to each clinical outcome.

#### 2.5.1. Predictor Variables

Two types of predictor variables were considered: clinical and pharmacogenetic. The clinical variables included in the analyses were:

- Gender (male/female);
- Age (years);
- Presence of other psychiatric comorbidities (yes/no);
- Presence of non-psychiatric comorbidities (yes/no);
- Smoking (number of cigarettes per day at enrolment);
- Alcohol consumption (number of standard units per day at enrollment);
- Previous bipolar episodes (number);
- Type of bipolar disorder (I or II);
- Overall CGI-BP score at the onset of the IE;
- Presence of suicidal ideation (yes/no);
- Use of anxiolytics (yes/no); and
- Presence of mood switch (yes/no; not included when switch was the predicted variable).

The pharmacogenetic variables included in the analysis were related to the following medications:

- Response to lithium (*CACNG2*);
- Reduced metabolism of quetiapine (*CYP3A4*);
- Variable metabolism of second-generation antipsychotics other than quetiapine (*CYP1A2*, *CYP2D6*);
- Lower side effects of risperidone or paliperidone (*AKT1-FCHSDQ-RPTOR-DDIT4*);
- Response to selective serotonin reuptake inhibitors (SSRIs) (*SLC6A4*, *BDNF*, *ABCB1*);
- Increased side effects of SSRIs (*SLC6A4*, *HTR2A*);
- Response to serotonin norepinephrine reuptake inhibitors (SNRIs) (*ABCB1*);
- Variable metabolism of SNRIs (*CYP2D6*);
- Response to anticonvulsants (*ABCB1*); and
- Increased side effects of anticonvulsants (*HLA-A*).

Pharmacogenetic variables were coded as +1 when the relevant genetic variant was detected in the genome of the patient and the patient was taking the particular affected drug, −1 when the genetic variant was not detected (i.e., the patient was wild-type for the polymorphism) and the patient was taking said affected drug, and 0 when the patient was not taking the affected drug, regardless of the patient’s genotype. Therefore, this codification system allowed to incorporate the aforementioned specific gene-drug interactions in the regression models, using the whole sample population (*n* = 76) per each gene-drug interaction evaluated.

#### 2.5.2. Clinical Outcomes

For the purposes of this study, long-term treatment response in bipolar depression was analyzed using the CGI-BP-M scores at enrollment (primary outcome measure) as the dependent variable. The score-change in this scale as a measure of treatment efficacy (i.e., patient improvement or worsening) was not assessed due to the lack of baseline scores for the HDRS-17 and FAST scales (secondary outcomes). To account for the possible effect of the severity of patients at the beginning of the disorder, the baseline CGI-BP-M score was considered as an independent variable in the statistical models, but it was not significant in any model.

Treatment tolerability was also assessed as a secondary outcome using the total number of adverse effects as a dependent variable in the regression models. Additionally, as autolytic ideation and mood switch could be characteristics of the disorder itself but also be driven by the pharmacological treatment, we chose to repeat the analysis adding these two factors to the definition of the dependent variable “adverse effects”.

All statistical analyses were performed with the application R studio version 1.2.1335 of *R* statistical software version 3.6.1 [35,36].

## 3. Results

### 3.1. Patient Characteristics

Sociodemographic and clinical characteristics of the study participants are summarized in Table 1. The evaluated subjects had a mean age of 43.5 ± 9.1 years, 65.8% were females, and 92.1% were of European descent. All patients had a diagnosis of BD with an index episode of major depression. A total of 53.9% of patients had BD type I and 44.7% BD type II. The mean age of onset was 31.2 ± 10.7 years, and the average duration of illness was 11.9 ± 8.6 years. Mood switch was experienced by 26.3% of the patients.

**Figure 2:**
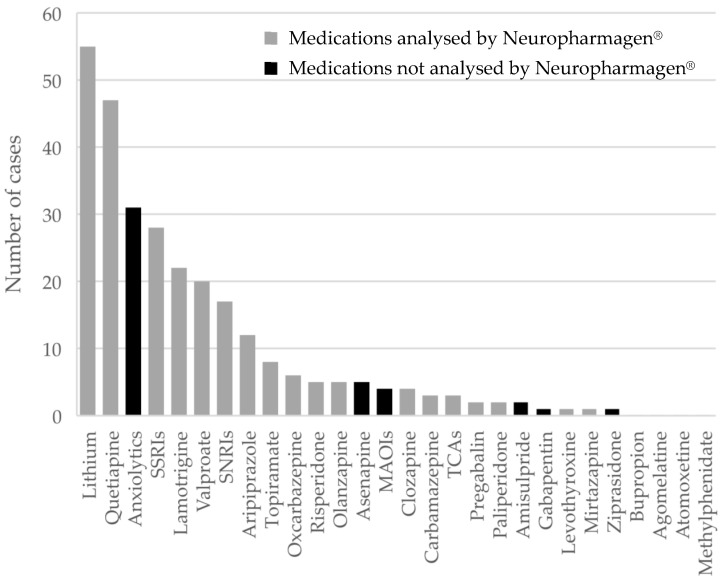
Medications for treating the index episode (*n* = 76). MAOI: Monoamine Oxidase Inhibitor; SNRI: Serotonin-Norepinephrine Reuptake Inhibitor; SSRI: Selective Serotonin Reuptake Inhibitor; TCA: Tricyclic Antidepressant.

Patients were prescribed an average of 4.5 ± 1.4 medications for their index episode. The most prescribed medications for treating the IE were lithium, lamotrigine, and valproate among mood stabilizers; selective serotonin reuptake inhibitors (SSRIs), and serotonin-norepinephrine reuptake inhibitors (SNRIs) among antidepressants; and quetiapine and aripiprazole among antipsychotics (Figure 2).

**Figure 3:**
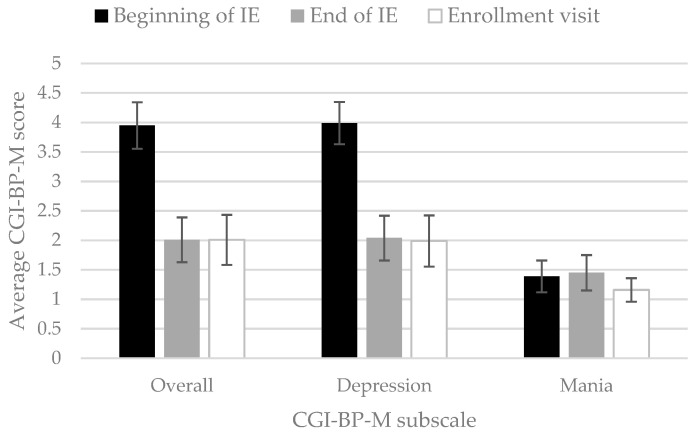
Clinical Global Impression for Bipolar Disorder modified version (CGI-BP-M) average scores (overall, depression and mania subscales) at the beginning of the index episode (IE) of major depression, end of IE, and at enrollment.

Baseline severity at the onset of the IE was evaluated using a modified version of the Clinical Global Impression for Bipolar Disorder (CGI-BP-M), yielding an average score of 3.9 ± 0.7 (overall), 4.0 ± 0.7 (depression subscale), and 1.4 ± 0.5 (mania subscale) (Table 2). At the end of the episode, a statistically significant improvement was observed in the CGI-BP-M depression and overall subscales (*p*-value < 0.001). This improvement was maintained at the enrollment visit (Figure 3).

### 3.2. Primary Results

Based on the premise that response to a pharmacological treatment, in terms of efficacy and/or tolerability, may be different when a medication is taken by a patient who carries a genetic polymorphism associated with efficacy, metabolism, and/or side effects, compared with a person with a wild-type genotype, multiple linear regression analysis was used to detect statistically significant associations between the clinical outcome of patients and the putatively predictive variables. Given the high number of drugs per case and the impossibility to assign a unique type of gene-drug interaction to each patient based on the PGx report, the analysis was focused on the effect of individual gene-drug pairs. Specifically, the analysis was conducted on those drugs for which, among patients taking them, there was a minimum of two cases with and without a particular pharmacogenetic variant.

The primary clinical outcome (dependent variable) analyzed was the CGI-BP-M score at enrollment. The CGI-BP-M score at the IE of major depression was considered as an independent variable in the statistical models to account for the possible effect of the severity of patients at beginning of the IE. The HDRS-17 score at enrollment and the number of adverse effects were included as secondary dependent variables in this study. Basic descriptive statistics and regression coefficients are shown in Table 3.

Pharmacogenetic variables that significantly correlated with the scores in the CGI-BP-M scale at enrollment were a response to SNRIs and reduced metabolism of quetiapine (Table 3). The genetically determined response to SNRIs—based on the analysis of the *ABCB1* gene—showed a significant negative correlation with the CGI-BP-M overall score (*p*-value < 0.030) and the depression subscore (*p*-value < 0.026) at enrollment, indicating an association with lower scores in both domains of this scale (Table 3). The response to SNRIs was also correlated with lower scores in the HDRS-17 scale at the time of analysis, and with a higher number of adverse effects (*p*-value < 0.024) (Table 3). The variable “reduced metabolism of quetiapine”—determined by the presence of CYP3A4*22 allele—was identified as a significant predictor of lower scores in the overall CGI-BP-M (*p*-value < 0.038) and the depression subdomain (*p*-value < 0.025) at enrollment. The pharmacogenetic variable lower risk of side effects of risperidone or paliperidone—based on the analysis of an mTOR-related multigenic predictor—was negatively correlated with the number of adverse effects, when including suicidal ideation and mood switch (*p* = 0.013)

Non-pharmacogenetic variables with a significant effect on the CGI-BP-M scores were the amount of tobacco and the treatment with anxiolytics (Table 3). The presence of comorbidities significantly predicted higher HDRS-17 scores. The number of previous episodes and the presence of psychiatric comorbidities were also positively correlated with the number of adverse effects.

## 4. Discussion

In recent years, substantial progress has been made in the field of pharmacogenetics in psychiatry. Particularly, several studies have demonstrated that pharmacogenetic-guided treatment significantly improves the efficacy and tolerability of medication in patients with a main diagnosis of major depressive disorder [37,38,39]. However, the clinical utility of pharmacogenetic testing for the treatment of BD has been less studied [40]. This observational retrospective study objectively evaluated the impact of the gene–drug pairs analyzed by a commercial pharmacogenetic-based tool in the response to pharmacological treatment in a well-characterized cohort of patients with BD [41].

As expected, the pharmacogenetic variable “response to SNRIs”, determined from the analysis of the genetic variation in the transporter gene *ABCB1* [42], was associated with lower scores in the CGI-BP-M overall and depression subscales (as well as the HDRS-17 scale), suggesting a role for this gene in predicting a good response to SNRI antidepressants in the context of BD treatment. These results are in accordance with previous studies linking genetic variation in *ABCB1* to the efficacy of antidepressant medications that are substrates of this protein [43,44,45,46]. Interestingly, in line with other studies, our results also suggested the association of *ABCB1* polymorphisms with an increased number of adverse effects [47,48]. The *ABCB1* gene—formerly known as the multi-drug resistance gene *MDR1*—codes for the drug efflux transporter permeability-glycoprotein (P-gp). P-gp actively drives the efflux of its substrates across cellular membranes in the intestine, liver, kidney, and the blood–brain barrier [49]. The presence of polymorphisms lowering the activity of this protein is thought to be linked with higher concentrations of its substrates in the brain, which may explain the increased likelihood of response, but also the poorer tolerability observed in our study.

The pharmacogenetic variable “reduced metabolism of quetiapine”, based on the analysis of the CYP3A4*22 allele, was associated with lower scores in the CGI-BP-M overall and depression subscales, thus positively impacting the likelihood of response. The CYP3A subfamily of enzymes is responsible for the metabolism of more than 50% of medications that undergo first-pass hepatic metabolism. The activity of CYP3A4 is highly variable due to changes in its expression levels that mainly occur in response to physiological states and environmental factors. Genetic variation has also been described to impact CYP3A4 activity. Particularly, the CYP3A4*22 polymorphism has been associated with reduced CYP3A4 expression levels and decreased enzyme activity in human livers [50,51]. Individuals carrying this variant have plasma levels of quetiapine 2.5 times higher than non-carriers at comparable doses [52]. Higher than expected serum concentrations of quetiapine in carriers of the CYP3A4*22 allele could explain a good response to this antipsychotic in our population. It is remarkable that the presence of this polymorphism did not correlate with an increased number of adverse effects in the studied cohort.

The genetic variable “lower side effects of risperidone or paliperidone” was correlated with a lower number of adverse effects when this outcome variable included switch and autolytical ideation. This genetic variable is based on the research carried out by Mas and collaborators [53], who developed a model for the prediction of antipsychotic-induced extrapyramidal symptoms through the analysis of the epistatic interactions of four genes of the DRD1-activated mTOR pathway (*AKT1*, *FCHSDQ*, *RPTOR*, and *DDIT4*). Surprisingly, this pharmacogenetic marker was associated with a much broader definition of adverse effects in our population, which warrants further studies.

Due to the observational uncontrolled design of this study, other available clinic-physiological characteristics of the patient sample were included in the statistical models to avoid the detection of false correlations with pharmacogenetic variables. Among non-pharmacogenetic variables associated with long-term patients’ status, the amount of tobacco and the treatment with anxiolytics were significantly positively correlated with the CGI-BP-M scores. The polycyclic aromatic hydrocarbons in cigarette smoke have been shown to induce CYP1A2, and different studies have reported decreased plasmatic concentrations of drugs primarily metabolized by CYP1A2 in smokers, including the antipsychotics clozapine and olanzapine [54,55], with the consequent poorer treatment response. We could not find a correlation between smokers with higher CGI-BP-M scores and treatment with clozapine or olanzapine, probably because of the small sample used in this study and the low number of patients treated with those drugs (*n* ≤ 5). Additionally, other patients’ characteristics, such as the severity of the disease itself may account for increased tobacco consumption. Likewise, treatment with anxiolytics, as a marker of anxiety in the study sample, was also associated with the long-term severity in our cohort. Finally, the number of adverse effects was found to be associated with the number of previous episodes and the presence of psychiatric comorbidities, which might be indirectly indicative of a higher number of medication trials or polymedication in these patients.

The predictors described in the present study could be of value for targeting patients with BD that most benefit from a particular medication in the long term. However, this study has some limitations. First, the reported R^2^ for the models obtained was between 0.14 and 0.33, meaning that 67% to 86% of the variation in the clinical outcomes assessed would not be explained by the models. Nevertheless, since the statistic is strongly influenced by variation in the independent variables, a small R^2^ may not necessarily be indicative of a weak relationship [56]. Additionally, a low R^2^ can be a good model in certain cases, such as in fields of study that have an inherently greater amount of variation, making it inevitable to obtain lower R^2^ values. This is the scenario found in the genetic study of many phenotypes. While in monogenic disorders such as cystic fibrosis or Duchenne muscular dystrophy a single gene/mutation is the cause of the disorder, complex disorders such as diabetes, cancer susceptibility, or psychiatric disorders themselves are known to be polygenic, being influenced by several genes/variants, each of them having a small contribution, and often in conjunction with environmental factors. Drug response is indeed a complex trait, affected by several pharmacokinetic and pharmacodynamic genes, but also by the biology of the treated disease itself and a number of environmental factors. It is in this context that the predictive value of the significant variables in this study is valuable, adding to the body of evidence of the clinical validity of these gene-drug interactions in bipolar disorder. Second, the high number of medications per patient in the study population made it impossible to assign each patient to only one response-related category (i.e., efficacy, metabolism, side effects, or standard/wild-type genotype) according to the Neuropharmagen^®^ report. Most patients were treated with different medications associated with different response categories and, in some cases, one medication was associated with more than one category. The frequency of the medications prescribed in the study population was markedly unequal: lithium, SSRIs, or quetiapine were used in many cases, while other psychotropic medications were much less used. In addition, the pharmacogenetic tool did not provide information for some of the medications used in some patients (black bars in Figure 3). All these factors, together with the small sample size, may have restricted the number of significant treatment predictors found in the study. Finally, although the cohort of patients studied is representative of what clinicians encounter in their usual practice, the retrospective observational design is an additional limitation of this exploratory analysis.

The results of this pilot study add to the evidence of the clinical validity of several gene–drug interactions in bipolar disorder (previously described to be relevant in other psychiatry conditions). However, further studies with larger samples of patients and a prospective, double-blind, randomized design will help determine if pharmacogenetic-guided treatment should become usual practice in the management of patients with BD.

## 5. Conclusions

Although studies proving the clinical utility of pharmacogenomics in BD are still in their early days, they offer a promise in improving clinical care. As more evidence becomes available, predictor models incorporating pharmacogenomics and clinical characteristics may one day help to decode the complexities of treatment responses in BD and successfully predict patient responses to medication.

## Figures and Tables

**Table 1:** Demographic and clinical characteristics of the study population.

Characteristic		*n* = 76
Gender, *n* (%)		
	Female	50 (65.78)
	Male	26 (34.21)
Ethnicity, *n* (%)		
	European	70 (92.10)
	Asian	1 (1.31)
	Hispanic	3 (3.94)
	Afro-American	1 (1.31)
	Others	1 (1.31)
Bipolar disorder type, *n* (%)		
	BD I	41 (53.94)
	BD II	34 (44.73)
	Data missing	1 (1.31)
Age at sampling (years), mean ± SD		43.54 ± 9.08
Age of onset of bipolar disorder (years), mean ± SD		31.16 ± 10.66
Duration of illness (years), mean ± SD		11.91 ± 8.64
Duration of index episodes (days), mean ± SD		76.91 ± 53.09
Number of drugs in index episode, mean ± SD		4.54 ± 1.43
Mood switch after index episode, *n* (%)		20 (26.31)
Number of adverse effects, mean ± SD		0.92 ± 1.03
Medical comorbidities, *n* (%)		22 (28.94)
	Non psychiatric comorbidities, *n* (%)	14 (18.42)
	Other psychiatric diagnostics, *n* (%)	14 (18.42)
Smoking, *n* (%)		40 (52.63)
Cigarettes per day, mean ± SD		10.03 ± 12.59
Alcohol intake, *n* (%)		11 (14.47)
Standard drink units per day, mean ± SD		0.34 ± 1.08
Substance use disorder, *n* (%)		5 (6.58)

BD I: Bipolar I Disorder; BD II: Bipolar II Disorder; SD: Standard Deviation.

**Table 2:** Clinical assessment of the study population.

		Time of Assessment	
Variable	Onset of the Episode	End of the Episode	Enrollment Visit
CGI-BP-M, mean ± SD			
Overall	3.95 ± 0.79	2.01 ± 0.76	2.01 ± 0.85
Depression	3.99 ± 0.72	2.04 ± 0.76	1.99 ± 0.87
Mania	1.39 ± 0.54	1.45 ± 0.60	1.16 ± 0.40
HDRS-17, mean ± SD	na	na	7.90 ± 6.04
FAST, mean ± SD	na	na	26.99 ± 19.04

CGI-BP-M: Clinical Global Impression for Bipolar Disorder, Modified version; FAST: Functioning Assessment Short Test; HDRS-17: 17-Item Hamilton Depression Rating Scale; na: Not assessed; SD: Standard Deviation.

**Table 3:** Multiple linear regression results between continuous variables selected by the forward-selection method and the clinical outcome assessed with the CGI-BP-M (overall and depression subscale), the HDRS-17 and the number of adverse effects.

Variables	B	SD	*β*	*t*	Sig.
**CGI-BP-M overall**					
R^2^ = 0.249; F = 5.898; *p*-value = 4 × 10^−4^					
(constant)	1.457	0.150		9.722	0.000
**Response to SNRIs**	**−0.454**	**0.205**	**−0.230**	**−2.215**	**0.030**
Smoke quantity	0.019	0.007	0.273	2.629	0.010
Treatment with anxiolytics in IE	0.457	0.181	0.263	2.529	0.014
**Reduced metabolism of quetiapine**	**−0.288**	**0.136**	**−0.221**	**−2.111**	**0.038**
**CGI-BP-M depression**					
R^2^ = 0.262; F = 6.303; *p*-value = 2 × 10^−4^					
(constant)	1.405	0.151		9.292	0.000
**Response to SNRIs**	**−0.472**	**0.207**	**−0.235**	**2.281**	**0.026**
Smoke quantity	0.020	0.007	0.290	2.816	0.006
Treatment with anxiolytics in IE	0.446	0.183	0.252	2.445	0.017
**Reduced metabolism of quetiapine**	**−0.315**	**0.138**	**−0.238**	**−2.292**	**0.025**
**HDRS-17**					
R^2^ = 0.326; F = 8.354; *p*-value = 0.000					
(constant)	-0.647	3.016		−0.215	0.831
**Response to SNRIs**	**−4.753**	**1.408**	**−0.336**	**−3.376**	**0.001**
Age at enrollment	0.137	0.071	0.205	1.929	0.058
Other psychiatric comorbidities	4.314	1.528	0.281	2.824	0.006
Medical comorbidities	3.025	1.399	0.230	2.162	0.034
**Number of adverse effects**					
R^2^ = 0.168; F = 6.664; *p*-value = 0.0023					
(constant)	0.731	0.152		4.809	0.000
Number of previous episodes	0.032	0.011	0.336	2.983	0.004
**Response to SNRIs**	**0.584**	**0.252**	**0.261**	**2.316**	**0.024**
**Number of adverse effects (including suicidal ideation and mood switch)**					
R^2^ = 0.138; F = 5.779; *p*-value = 0.0047					
(constant)	**1.576**	**0.126**		**12.531**	**0.000**
**Lower side effects of risperidone or paliperidone**	**−1.115**	**0.439**	**−0.278**	**−2.539**	**0.013**
Other psychiatric comorbidities	**0.638**	**0.291**	**0.240**	**2.197**	**0.031**

Pharmacogenetic variables in bold. B: Unstandardized regression coefficient; SD: Standard Deviation; *t*: Student’s *t*; *β*: Standardized regression coefficient; Sig: Significance (*p* value < 0.05); R^2^: Coefficient of determination.

## Data Availability

Not applicable.

## Supplementary Materials

The following are available online at https://www.mdpi.com/article/10.3390/genes12081259/s1, Figure S1: Example of a Neuropharmagen^®^ pharmacogenomics interpretative report for one de-identified study subject, showing (A) the color-coding classification of drugs, and (B) detailed information for one of the drugs, Table S1: List of genes and polymorphisms analyzed.
