# Supplementary material for: Genetic Variations Associated with Long-Term Treatment Response in Bipolar Depression
Source: Genes (Basel). 2021 Aug 18;12(8):1259. doi: 10.3390/genes12081259 (PMC8391230; doi:10.3390/genes12081259)
Supplement: Supplementary file 1 [file genes-12-01259-s001.zip › genes-1310708-supplementary.pdf]

genes

MDPI

Figure S1. Example of a Neuropharmagen® pharmacogenomics interpretative report for one de-identified study subject, showing (**A**) the color-coding classification of drugs, and (**B**) detailed information for one of the drugs.

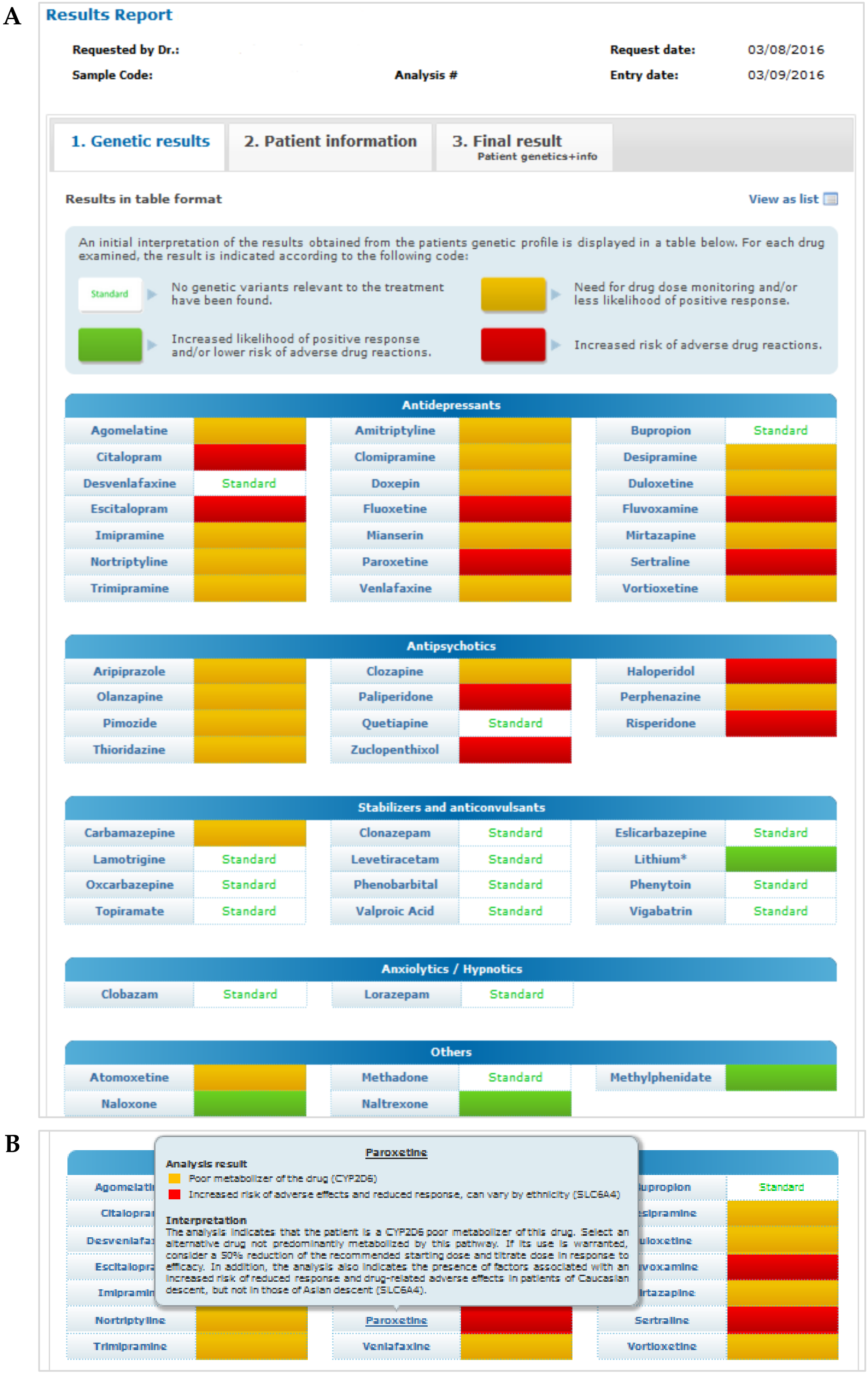

Table S1. List of genes and polymorphisms analyzed

**Gene symbol**	**Gene name**	**Polymorphisms**
ABCB1	ATP binding cassette subfamily B member 1	rs2235048, rs11983225
AKT1	V-akt murine thymoma viral oncogene homolog 1	rs1130214
BDNF	Brain-derived neurotrophic factor	rs6265
CACNG2	Calcium channel, voltage-dependent, gamma subunit 2	rs2284017
CES1	Carboxylesterase 1	rs71647871
COMT	Catechol-O-methyltransferase	rs4680
CRHR1	Corticotropin releasing hormone receptor 1	rs4792888
CYP1A2	Cytochrome P450 family 1 subfamily A member 2	*1, *1F
CYP2B6	Cytochrome P450 family 2 subfamily B member 6	*1, *6
CYP2C19	Cytochrome P450 family 2 subfamily C member 19	*1, *2, *3, *4, *5, *6, *7, *8, *9, *10, *17, *27
CYP2C9	Cytochrome P450 family 2 subfamily C member 9	*1, *2, *3, *5, *6, *8, *11, *27
CYP2D6	Cytochrome P450 family 2 subfamily D member 6	*1, *2, *3, *4, *5, *6, *7, *8, *9, *10, *11, *12, *14, *15, *17, *19, *20, *29, *30, *35, *36, *36-*10 hybrid tandems, *40, *41, *69, gene amplifications *1xN, *2xN, *4xN, *10xN, *41xN, and gene duplications *3x2, *6x2, *9x2, *17x2, *29x2, *35x2
CYP3A4	Cytochrome P450 family 3 subfamily A member 4	*1, *22
DDIT4	DNA damage inducible transcript 4	rs1053639
DRD3	Dopamine receptor D3	rs963468
EPHX1	Epoxide hydrolase 1, microsomal (xenobiotic)	rs1051740
FCHSD1	FCH and double SH3 domains 1	rs456998
GRIK2	glutamate receptor, ionotropic, kainate 2	rs2518224
GRIK4	glutamate receptor, ionotropic kainate 4	rs1954787
HLA-A	Major histocompatibility complex, class I, A	rs1061235
HTR1A	5-HT (serotonin) receptor 1A, G protein-coupled	rs10042486
HTR2A	5-HT (serotonin) receptor 2A, G protein-coupled	rs6311, rs6314, rs9316233
HTR2C	5-HT (serotonin) receptor 2C, G protein-coupled	rs1414334
LPHN3	Latrophilin 3	rs6551665
NEFM	Neurofilament, medium polypeptide	rs1379357, rs1457266
OPRM1	Opioid receptor, mu 1	rs1799971
RGS4	Regulator of G-protein signaling 4	rs2661319
RPTOR	Regulatory associated protein of MTOR, complex 1	rs7211818
SLC6A4	Solute carrier family 6 (neurotransmitter transporter), member 4	5-HTTLPR
UGT2B15	UDP glucuronosyltransferase 2 family, polypeptide B15	rs1902023

Copyright: © 2021 by the authors. Submitted for possible open access publication under the terms and conditions of the Creative Commons Attribution (CC BY) license (https://creativecommons.org/licenses/by/4.0/).
